# Antitumor Activity of Asperphenin A, a Lipopeptidyl Benzophenone from Marine-Derived *Aspergillus* sp. Fungus, by Inhibiting Tubulin Polymerization in Colon Cancer Cells

**DOI:** 10.3390/md18020110

**Published:** 2020-02-13

**Authors:** Song Yi Bae, Lijuan Liao, So Hyun Park, Won Kyung Kim, Jongheon Shin, Sang Kook Lee

**Affiliations:** College of Pharmacy, Natural Products Research Institute, Seoul National University, Seoul 08826, Korea; baesy722@gmail.com (S.Y.B.); liao_0909@126.com (L.L.); hanirela@snu.ac.kr (S.H.P.); dnjsrud6764@naver.com (W.K.K.)

**Keywords:** marine microorganism metabolite, *Aspergillus* sp. fungus, asperphenins, colon cancer, antimitotic agent, tubulin polymerization

## Abstract

Marine-derived microorganisms are a valuable source of novel bioactive natural products. Asperphenin A is a lipopeptidyl benzophenone metabolite isolated from large-scale cultivation of marine-derived *Aspergillus* sp. fungus. The compound has shown potent antiproliferative activity against various cancer cells. However, the underlying mechanism of action remained to be elucidated. In this study, we demonstrated the antitumor activity and molecular mechanism of asperphenin A in human colon cancer cells for the first time. Asperphenin A inhibited the growth of colon cancer cells through G_2_/M cell cycle arrest followed by apoptosis. We further discovered that asperphenin A can trigger microtubule disassembly. In addition to its effect on cell cycle, asperphenin A-induced reactive oxygen species. The compound suppressed the growth of tumors in a colon cancer xenograft model without any overt toxicity and exhibited a combination effect with irinotecan, a topoisomerase I inhibitor. Moreover, we identified the aryl ketone as a key component in the molecular structure responsible for the biological activity of asperphenin A using its synthetic derivatives. Collectively, this study has revealed the antiproliferative and antitumor mechanism of asperphenin A and suggested its possibility as a chemotherapeutic agent and lead compound with a novel structure.

## 1. Introduction

Bioactive molecules isolated from nature have largely contributed to novel drugs or lead compounds for various human diseases. Therefore, many drugs currently used in the clinic are natural products or their derivatives [1,2]. Among the diverse sources of natural products, marine-derived microorganisms are widely known to produce a large number of biologically active metabolites with very unique structures due to their distinctive habitats, isolation, and culture conditions [3]. This promotes the opportunity to discover the chemicals of novel classes with therapeutic potential. Several studies have reported that these metabolites exhibit various bioactivities, such as antimicrobial, anti-inflammatory, antiviral, and anticancer activities [4].

The cell cycle consists of five distinct phases: quiescence (G_0_), Gap 1 (G_1_), DNA replication/synthesis (S), Gap 2 (G_2_), and mitosis (M). Cells progress through the cell cycle under tight regulation by checkpoints, however, often these controls are lost in cancer [5,6,7]. Therefore, anticancer agents directly or indirectly act on the process of the cell cycle to terminate the unusual growth of cancer cells. One of the most common and yet robust mechanisms to arrest cell cycle progression is targeting microtubules, which disrupts the polymerization dynamics of mitotic spindles during mitosis and leads to cell death [8]. A large number of natural compounds and their analogs are classified as antimitotic agents and used in the treatment of cancer [9,10]. Some peptides from marine organisms have been also identified to interfere with mitotic spindle formation [11,12,13,14,15].

In our previous study, we have isolated and identified asperphenins A (AspA) and B (AspB), lipopeptidyl benzophenone metabolites of a novel skeletal class, from a marine-derived *Aspergillus* sp. fungus [16]. The structure of asperphenins consists of a hydroxy fatty acid, a tripeptide, and a trihydroxybenzophenone. These are epimers with an opposing absolute configuration at C-17. Both compounds inhibited the growth of various cancer cell lines, suggesting their therapeutic potential as anticancer agents. Here, we investigated the antiproliferative mechanism of AspA in a colon cancer cell line for the first time. In the present study, we found that AspA induces cell death via G_2_/M phase arrest by inhibition of tubulin polymerization. The antitumor effect of AspA in a colon cancer xenograft model and a combined treatment with other chemotherapeutic agents further supported its possibility to be developed as a novel anticancer drug. In addition, the structure-activity relationship study using analogs of AspA confirmed a moiety essential for biological activity, which allows the development of potent derivatives in this novel skeletal class. These findings suggest AspA can be a new lead compound in the discovery of chemotherapeutic agents with antimitotic activity.

## 2. Results

### 2.1. Aryl ketone at C-7 is Responsible for the Antiproliferative Effect of Asperphenins

**Figure 1:**
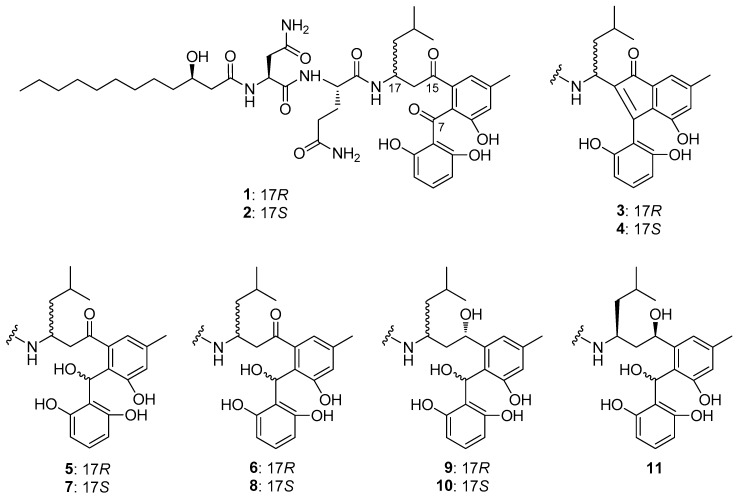
Structures of asperphenins and synthetic derivatives: Asperphenin A (**1**); asperphenin B (**2**); cycloasperphenin A (**3**); cycloasperphenin B (**4**); 7-hydroxyasperphenin A (**5**); 7-*epi*-hydroxyasperphenin A (**6**); 7-hydroxyasperphenin B (**7**); 7-*epi*-hydroxyasperphenin B (**8**); 7, 15(*S*)-dihydroxyasperphenin A (**9**); 7,15(*S*)-dihydroxyasperphenin B (**10**); 7,15(*R*)-dihydroxyasperphenin B (**11**).

We previously reported the potent antiproliferative activities of asperphenins A (AspA) and B (AspB) against a panel of human cancer cell lines, including RKO, SNU638, SK-HEP-1, and MDA-MB-231 cells [16]. To further determine the structure-activity relationship, the cell growth inhibition effects of synthetic derivatives (Figure 1; [16]) were evaluated in the same panel of cell lines. The IC_50_ values of derivatives either increased more than 5-fold or were unmeasurable (Table 1, Figure S1). All of the synthetic derivatives with a reduced aryl ketone at C-7 to hydroxyl or olefinic group exhibited negligible antiproliferative activity, indicating the aryl ketone moiety as a critical group in the molecular structure required for the cytotoxic effect of asperphenins.

### 2.2. AspA Induces G_2_/M Phase Cell Cycle Arrest by Inhibiting Microtubule Assembly

**Figure 2:**
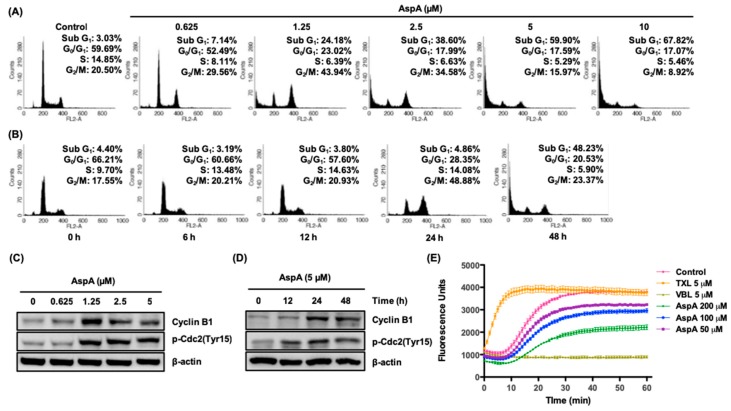
Effect of AspA on cell cycle progression and tubulin polymerization in RKO cells. (**A**,**B**) Cells were treated with the indicated concentrations of AspA for 48 h (**A**) or 5 μM of AspA for indicated times (**B**) and then analyzed by flow cytometry. (**C**,**D**) The levels of G_2_/M regulatory proteins in cells were determined after treatment with various concentrations of AspA for 48 h (**C**) or 5 μM of AspA over time (**D**) by western blot analysis. β-Actin was used as a loading control. (**E**) Inhibitory effect of AspA on tubulin polymerization. The tubulin assembly in the presence of vehicle or indicated compounds was analyzed by measuring the fluorescence emitted at 450 nm using 360 nm as an excitation wavelength for 60 min at 37 °C. Paclitaxel (TXL) and vinblastine (VBL) were used as reference compounds. The data are presented as means ± SD.

As the potency of AspA in antiproliferative activity is known (ref [16]; 0.8 μM in RKO colon cancer cells), the mechanism of action was explored in RKO colon cancer cells. Deregulation of the cell cycle causes abnormal cell proliferation in cancer [5]. Therefore, to investigate whether AspA affects cell cycle progression in RKO cells, the cell cycle distribution was assessed by flow cytometry. In response to treatment with AspA for 48 h, a marked accumulation of cells in the sub-G_1_ apoptotic phase was detected in a concentration-dependent manner (Figure 2A). In addition, the percentage of cells in G_2_/M phase increased from 20.50% (Control) to 43.94% (1.25 μM). Moreover, the time-course observation of cells treated with 5 μM AspA exhibited that G_2_/M phase arrest was predominant for up to 24 h, followed by apoptosis (Figure 2B). The observed changes in cell cycle progression were confirmed by evaluating the expression of G_2_/M regulatory proteins, cyclin B1, and cdc2 in AspA-treated RKO cells. Consistent with the observed increase in G_2_/M phase cell-population, the expression of cyclin B1 was elevated after treatment with AspA for 48 h or time course treatment with 5 μM AspA (Figure 2C,D). A slight reduction of cyclin B1 levels was observed with 2.5 and 5 μM AspA treatment compared to 1.25 μM treated cells. This may be due to a strong shift in cell cycle distribution towards sub-G_1_. Active cyclin B1/cdc2 complex is essential in the transition from G_2_ phase to mitosis, and the activity of the complex is regulated by inhibitory phosphorylation of cdc2 at Tyr15 [17]. The expression of p-cdc2 (Tyr15) was increased in a concentration- and time-dependent manner, as indicative of decreased cyclin B1/cdc2 complex activity (Figure 2C,D). Suppression of microtubule dynamics by microtubule targeting agents induces mitotic arrest and trigger cell death [18]. Therefore, we evaluated whether AspA is capable of modulating tubulin polymerization using a cell-free in vitro assay. As a result, the compound inhibited tubulin polymerization in a similar manner of vinblastine (VBL) (Figure 2E). The percentage of polymerization inhibition was 15%, 22%, and 42% for 50, 100, and 200 μM AspA, respectively. Collectively, these data suggest that AspA exhibits antiproliferative activity through modulation of tubulin polymerization, which leads to G_2_/M phase arrest and apoptosis in RKO cells.

### 2.3. AspA Treated-Cells Undergo Apoptosis and Produce Intracellular Reactive Oxygen Species

**Figure 3:**
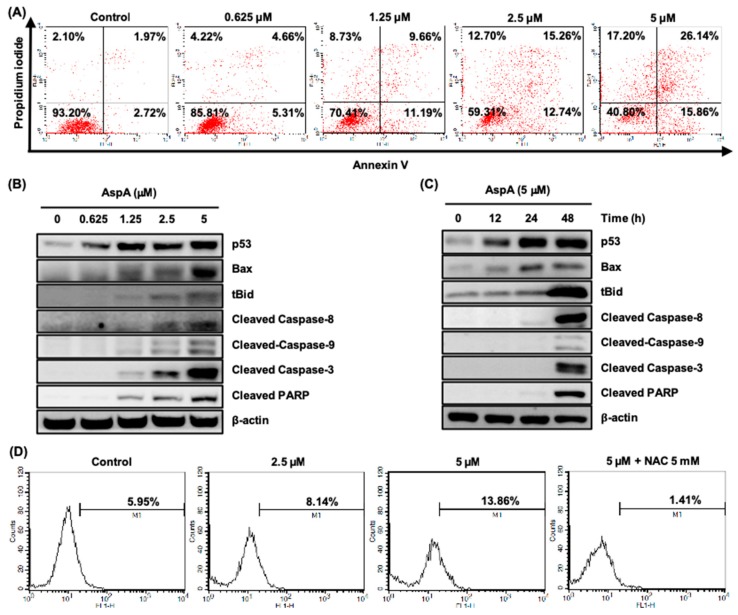
Effect of AspA on the induction of apoptosis and intracellular ROS in RKO cells. (**A**) Cells were treated with the indicated concentrations of AspA for 48 h. Collected cells were stained with Annexin V-FITC and PI, and analyzed by flow cytometry. (**B**,**C**) The expression of apoptosis-associated proteins was observed in cells treated with the indicated concentrations of AspA for 48 h (**B**) or 5 μM of AspA for the indicated times (**C**) by western blot analysis. β-Actin was used as a loading control. tBid, truncated Bid. (**D**) Cells were treated with AspA alone or with 5 mM of NAC for 24 h. The cells were stained with DCFH-DA and then analyzed by flow cytometry.

Cells arrested in mitosis eventually undergo apoptosis during mitotic arrest or exit mitosis, which is followed by cell death (via apoptosis or not), cell senescence and aneuploidy [19]. As shown in Figure 2A,B, AspA strongly induced the Sub-G_1_ cell population after the mitotic arrest. Therefore, we performed Annexin V-FITC and PI dual staining to confirm the induction of cell death. The compound treatment for 48 h led to an increase in early and late apoptosis, indicated by Annexin V-positive and Annexin V-PI dual positive, respectively (Figure 3A). Tumor suppressor p53 is critically involved in apoptotic cell death through modulation of Bcl-2 family members and activation of caspases and poly (ADP-ribose) polymerase (PARP) [20]. A concentration- and time-dependent activation of p53, pro-apoptotic Bcl-2 family member Bax and Bid, and cleavage of caspase-8, 9, 3 and PARP were detected in RKO cells treated with AspA, indicating the trigger of both intrinsic and extrinsic apoptotic pathways (Figure 3B,C). Moreover, AspA-mediated activation of caspases and PARP were prominent at 48 h (Figure 3C), which corresponds to Figure 2B. Some antimitotic agents, such as paclitaxel, vincristine, and taxotere, are also known to induce cell death by generating reactive oxygen species (ROS), which also leads to the cytotoxic bystander effect [21,22]. Thus, we assessed whether AspA promotes intracellular ROS with DCFH-DA staining using flow cytometry. As shown in Figure 3D, the intracellular ROS level in RKO cells increased in a concentration-dependent manner after 24 h treatment with AspA. In addition, the induction of ROS by AspA was reduced by co-treatment with a free radical scavenger, N-acetylcysteine (NAC). These results suggest that AspA-mediated cell death occurs through the sequential process of mitotic arrest and apoptosis, and the production of ROS further enhances the apoptotic cell death.

### 2.4. AspA Enhances the Effect of Irinotecan on Cell Growth Inhibition

**Figure 4:**
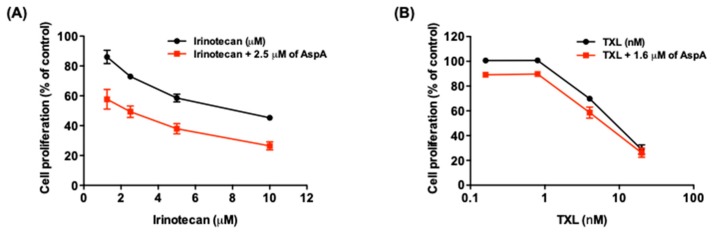
Effect of combined treatment with AspA and irinotecan or paclitaxel (TXL) on cell proliferation in RKO cells. (**A**,**B**) Cells were treated with the indicated concentrations of either irinotecan (**A**) or TXL (**B**) with or without AspA for 48 h. The cell proliferation was measured by SRB assay and the combination effect was determined by calculating combination index (CI) values. The data are presented as means ± SD.

To explore the potential use of AspA in combination with conventional chemotherapeutic drugs, the combination effect of AspA with a topoisomerase I inhibitor, irinotecan, was assessed in RKO cells. Irinotecan is a key component of standard treatment regimens for metastatic colon cancer [23]. The combined treatment demonstrated a synergistic effect on suppressing the growth of RKO cells (Figure 4A, Table 2). Additionally, AspA was evaluated for a combination effect with paclitaxel, which is an antimitotic agent. As a result, AspA did not enhance the antiproliferative activity of paclitaxel (Figure 4B, Table 3). Although the concentration of AspA used in the combination with paclitaxel was lower than that with irinotecan, the result may be due to the contrasting effect of two compounds on tubulin polymerization; AspA inhibits microtubule assembly, whereas paclitaxel inhibits disassembly. Taken together, AspA enhances the antiproliferative effect of irinotecan, which has a distinct mechanism of action from AspA.

### 2.5. AspA Suppresses the Tumor Growth in RKO Cells-Implanted Nude Mouse Xenograft Models

**Figure 5:**
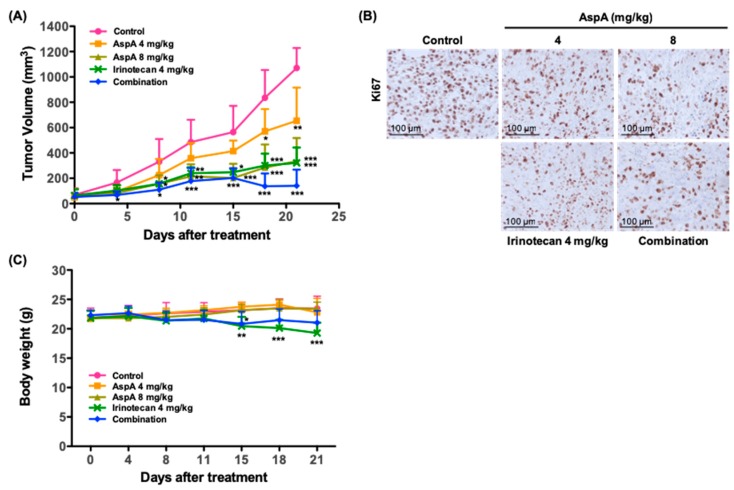
Antitumor activity of AspA in a nude mouse xenograft model. (**A**) RKO xenograft-bearing nude mice were treated intraperitoneally with the indicated drugs three times per week for 21 days (n = 5 per group). For combination, 4 mg/kg of AspA and 4 mg/kg of irinotecan were administered. Tumor volumes were measured every 3–4 days. (**B**) Ki67 protein expression in RKO cell xenograft tumors was determined by immunohistochemical analysis. Scale bar, 100 μm. (**C**) The bodyweight of mice bearing RKO xenografts was measured every 3–4 days during the treatment with the indicated drugs (n = 5 per group). The error bars represent the means ± SD. * *P* < 0.05, ** *P* < 0.01, *** *P* < 0.005 by *t*-test.

The in vitro antiproliferative effect of AspA was further evaluated in nude mouse tumor xenograft models implanted with RKO cells. Treatment with 4 or 8 mg/kg of AspA significantly inhibited the tumor growth by 38.9% and 68.7%, respectively, at the end of the study (Figure 5A, Table 4). Consistent with the in vitro findings, the combination of AspA with irinotecan demonstrated a moderate synergistic effect on suppressing the tumor growth (Figure 5A, Table 5). Antitumor activity of the compound was further supported by the decreased staining of Ki-67, a cellular proliferation marker, in tumor sections from groups treated with AspA alone and a combination of AspA and irinotecan (Figure 5B). A group treated with irinotecan alone was assessed in parallel. As expected, irinotecan showed strong inhibition of tumor growth and Ki-67 expression (Figure 5A,B). No overt toxicity or body weight change was observed in AspA-treated groups, whereas reduced body weight was detected in irinotecan-treated groups (Figure 5C). Taken together, AspA alone and co-treatment with irinotecan effectively suppressed the growth of RKO xenograft tumors. Although further experiments are needed, AspA may help reduce the toxicity of irinotecan by lowering its dose but maintaining the equivalent antitumor activity.

## 3. Discussion

Despite the complexity in cultivating and identifying bioactive metabolites from marine-derived microorganisms, it has been a valuable source for a tremendous number of diverse therapeutic compounds due to the biodiversity of the marine environment [3,24]. In our previous work, we reported two compounds, which showed antiproliferative activities against human cancer cell lines, from a strain of *Aspergillus* collected from marine-submerged decaying wood [16]. The compounds were asperphenins A and B with novel diastereomeric lipopeptidyl benzophenone structures. Here, we conducted a subsequent study to elucidate the detailed antiproliferative mechanism of asperphenins by applying asperphenin A in the RKO human colon cancer cell line, which was the most sensitive cancer cell line to both compounds with the similar IC_50_ values.

The present study demonstrated that the antiproliferation activity of asperphenin A corresponds to the regulation of the cell cycle. The observation of asperphenin A-mediated changes in cell cycle and related protein markers revealed that the compound inhibits the growth of colon cancer cells through two consecutive cellular events: G_2_/M cell cycle arrest followed by apoptosis. Drugs inducing G_2_/M cell cycle arrest often interfere with the function of microtubules to inhibit cell proliferation and subsequently result in cell death [18,25]. Microtubules play a significant role in cell growth, division, motility, morphology, and intracellular trafficking [26]. Therefore, stabilizing or destabilizing microtubule polymerization is an effective approach in cancer treatment. In this study, we utilized a cell-free fluorescence-based tubulin polymerization assay to assess the capability of asperphenin A to modulate tubulin assembly. We report for the first time that asperphenin A can prevent microtubule polymerization. Although further studies identifying the binding sites of asperphenin A on tubulin are required, the findings strongly suggest the potential of asperphenin A as a microtubule-destabilizing agent. Furthermore, the antagonistic effect of drug combination with paclitaxel, a microtubule-stabilizing agent, indirectly supports the opposing molecular mechanism of two agents.

Several studies have shown that chemotherapeutics promote intracellular levels of reactive oxygen species (ROS) as a mechanism to kill cancer cells [27]. However, the role of ROS in cancer cells is controversial that it is considered as a tumor-suppressing and promoting agent [28,29]. Despite the conflicting impact of ROS in the tumor, the removal of ROS by antioxidants increased tumorigenesis and decreased survival in clinical trials [30,31,32]. Modulating ROS levels can also sensitize multidrug-resistant cancer cells to chemotherapeutics [33]. Moreover, some antimitotic agents are reported to elevate ROS levels and cause damage to neighboring cancer cells [21,22]. Likewise, we showed that asperphenin A also induces intracellular ROS contributing to its apoptotic effect. To further ensure the tumor-suppressing effect of ROS induced by asperphenin A, an extensive study will be evaluated.

Combination with irinotecan, a topoisomerase I inhibitor and one of the first-line treatments for metastatic colon cancer [23], resulted in the enhanced antiproliferative and antitumor activity of asperphenin A. According to the bodyweight measurements of xenograft-bearing mice, we noticed significant bodyweight reduction in the irinotecan-treated group, while no changes were observed in asperphenin A-treated group. This suggests the possibility of applying asperphenin A in combination with chemotherapeutic agents possessing toxicity to obtain the successful antitumor effect and yet minimize the toxicity.

From the structure-activity relationship study by synthetic derivatives, we defined the aryl ketone moiety as a cytotoxic group of asperphenins. The identified bioactive group enables further development of asperphenins analogs that may exhibit better properties in terms of antitumor activity, bioavailability and solubility, etc. Due to its novel structure, a distinct class of antimitotic agents may be built from asperphenin A. Moreover, the feasible large-scale cultivation of *Aspergillus* strain allows one to overcome the limitation in compound supply from a natural source, which most nature-derived drug candidates encounter [34,35].

Collectively, this study has revealed the antiproliferative and antitumor mechanism of asperphenin A and provided evidence of its possibility as a promising new lead compound in the discovery of chemotherapeutic agents.

## 4. Materials and Methods

### 4.1. Preparation of Asperphenins and Synthetic Derivatives

Asperphenins A (**1**) and B (**2**) were isolated from a semi-solid culture broth of a marine-derived *Aspergillus* sp. fungus [16]. These were converted to cycloasperphenins A (**3**) and B (**4**), respectively, by treatment with K_2_CO_3_ in DMF. Compound **1** was also readily reduced to epimeric 7-hydroxy derivatives **5** and **6** with NaBH_4_ in MeOH. Further reduction of **5** provided two epimeric 7,15-dihydroxy derivatives **9** and an unreported one. Similarly, compound **2** was reduced to 7,15-dihydroxy derivatives **11** and **12** via the 7-hydroxy derivative **7**. Based upon the results of combined ^1^H and ^13^C NMR analyses, **5** and **7** were found to possess the same configuration at C-7 while **6** and **8** possessed the opposite one. Due to the severe steric crowding at this stereogenic center, however, further configurational assignments at C-7 were unaccomplished. In addition, it would be noteworthy that the epimeric 7-hydroxy derivatives **6** and **8** from **1** and **2**, respectively, were completely decomposed during the second-phase reduction at C-15, possibly due to the steric interactions of the 7-hydroxy group with the newly formed 15-hydroxy group. The absolute configurations at all of the stereogenic centers of asperphenins and synthetic derivatives (besides C-7) were assigned by combined MTPA methods, *J*-based analyses and ECD calculations [16].

### 4.2. Cell Proliferation Assay

Human gastric carcinoma (SNU-638), hepatocellular adenocarcinoma (SK-HEP-1), and breast adenocarcinoma (MDA-MB-231) cells were purchased from the Korean Cell Line Bank (Seoul, Korea). Colorectal carcinoma (RKO) cells were purchased from the American Type Culture Collection (Manassas, VA, USA). Cells were cultured in medium (DMEM for RKO, SK-HEP-1, and MDA-MB-231 cells; RPMI 1640 for SNU-638 cells) supplemented with 10% FBS and antibiotics-antimycotics (PSF; 100 units/mL penicillin G sodium, 100 μg/mL streptomycin, and 250 ng/mL amphotericin B) at 37 °C and 5% CO_2_ in a humidified atmosphere. Cells were seeded at a density of 3–7 × 10^4^ cells/mL in 96-well culture plates, and then treated with indicated compounds for 72 h. At the end of the experiment, cells were fixed with 10% TCA solution and subjected to sulforhodamine B (SRB) assay to determine cell proliferation [36]. The percentage of cell proliferation was calculated with the following formula:

Cell proliferation (%) = 100 × [(A_treated_ − A_zero day_)/(A_control_ − A_zero day_)],

where A is the average absorbance. The IC_50_ values were calculated through non-linear regression analysis using TableCurve 2D v5.01 (Systat Software Inc., San Jose, CA, USA). All experiments were performed in triplicate and data shown are representative of two or three independent experiments.

### 4.3. Cell Cycle Analysis

RKO cells were seeded on a 100-mm culture dish and incubated with various concentrations of AspA for the indicated times. Following the incubation, the cells were harvested and fixed with 70% ethanol overnight at 4 °C. After washing with PBS, the fixed cells were incubated with a staining solution containing RNase A (50 μg/mL) and propidium iodide (50 μg/mL) in PBS for 30 min at room temperature. The prepared samples were analyzed on a FACSCalibur^®^ flow cytometer (BD Biosciences, San Jose, CA, USA). A minimum of 10,000 events was collected for each analysis, and DNA histograms were generated and analyzed using CELLQuest^TM^ Pro software (BD Biosciences). Data shown are representative of two independent experiments.

### 4.4. Western Blot Analysis

To prepare total cell lysates, the cells were lysed in 2× sample lysis buffer (250 mM Tris-HCl pH 6.8, 4% SDS, 10% glycerol, 0.006% bromophenol blue, 2% β-mercaptoethanol, 50 mM sodium fluoride, and 5 mM sodium orthovanadate). Protein concentrations were determined by BCA protein assay, and equal amounts of proteins were subjected to sodium dodecyl sulfate-polyacrylamide gel electrophoresis. Separated proteins were transferred onto polyvinylidene difluoride membranes (Millipore, Bedford, MA, USA) and probed with appropriate primary and secondary antibodies. The blots were exposed to enhanced chemiluminescence solution (Intron, Daejon, Korea) and detected with an LAS-4000 (Fuji Film Corp., Tokyo, Japan). Data shown are representative of three independent experiments.

### 4.5. Tubulin Polymerization Inhibition Assay

The tubulin polymerization was evaluated by fluorescence-based assay according to the manufacturer’s instructions (Cytoskeleton Inc., Denver, CO, USA). Porcine tubulin (2 mg/mL) in 1× buffer (80 mM PIPES pH 6.9, 2 mM MgCl_2_, 0.5 mM EGTA, and 10 μM fluorescent reporter) containing 1 mM GTP and 20% glycerol was incubated with 1% DMSO (vehicle) or indicated concentrations of compounds in a 96-well plate pre-warmed to 37 °C. The tubulin polymerization reactions were observed by recording fluorescence emitted at 450 nm using 360 nm as an excitation wavelength for 60 min at 37 °C with a SpectraMax M5 plate reader (Molecular Devices, Sunnyvale, CA, USA). The percentage of polymerization inhibition was determined as follows:

Inhibition of tubulin polymerization (%) = 100 × (1 − FU_compound_/FU_vehicle_),

where FU is the average fluorescence unit for compounds or vehicle at the end of the reactions. Paclitaxel and vinblastine were used as reference compounds. All experiments were performed in duplicate and the data shown are representative of two independent experiments.

### 4.6. Annexin V/PI Staining

RKO cells were treated with various concentrations of AspA for 48 h. The cells were trypsinized for collection and washed twice with cold PBS. Cells were then incubated with 1× binding buffer containing Annexin V-FITC and PI for 15 min at room temperature in the dark. The stained cells were analyzed using a FACSCalibur^®^ flow cytometer (BD Biosciences). Data shown are representative of two independent experiments.

### 4.7. DCFH-DA Staining

RKO cells were seeded in 6-well culture plates overnight and then treated with AspA with or without 5 mM NAC for 24 h. The cells were stained with 20 μM DCFH-DA for 30 min followed by analysis with a FACSCalibur^®^ flow cytometer (BD Biosciences). Data shown are representative of two independent experiments.

### 4.8. In Vitro Drug Combination Analysis

RKO cells were cultured in 96-well plates and then exposed to various concentrations of irinotecan or paclitaxel with AspA in a ratio of 1:1. After 48 h incubation, cell proliferation was examined by the SRB assay. The combination effect was evaluated by calculating the combination index (CI) values with the following formula:

CI = D_1_/(D_x_)_1_ + D_2_/(D_x_)_2_, (1)

where D_1_ and D_2_ are the concentrations of the combined test compounds that achieve the expected effect, and (D_x_)_1_ and (D_x_)_2_ are the concentrations that achieve similar effects when the test compounds are used alone. In the present study, 50% inhibition was taken as the effective level. The CI values were compared to the reference values reported by Chou [37]. All experiments were performed in triplicate and data shown are representative of two independent experiments.

### 4.9. In Vivo Tumor Xenograft Model

All animal use and care protocols followed the guidelines of the Institutional Animal Care and Use Committee at Seoul National University and were approved by the Korean Association of Laboratory Animal Care. RKO cells were injected subcutaneously into the flanks of the mice (3.5 × 10^6^ cells in 150 μL of medium). When the tumors reached a volume of 60 mm^3^, the mice were randomly assigned to the vehicle control and treatment groups (n = 5 per group). AspA (4 or 8 mg/kg) or irinotecan (4 mg/kg) dissolved in a volume of 300 μL of vehicle solution (0.5% Tween 80 in normal saline) was administered intraperitoneally three times per week for 21 days. The combination group was treated with 4 mg/kg of AspA and 4 mg/kg of irinotecan. The control group was treated with an equal volume of vehicle. The tumor size was measured using a digital slide caliper and volumes (mm^3^) were determined as follows:

Tumor volume (mm^3^) = (width) × (length) × (height) × π/6.

The percentage of tumor growth inhibition was estimated as follows:

Inhibition of tumor growth (%) = 100 × [1 − (TV_f, treated_ − TV_i, treated_)/(TV_f, control_ − TV_i, control_)],

where TV_f_ is the average tumor volume at the end of the study, and TV_i_ is the average tumor volume at Day 0, the day before the first administration. The body weight of each mouse was also monitored for toxicity.

### 4.10. In Vivo Drug Combination Analysis

The synergistic effect of AspA and irinotecan was analyzed on RKO xenograft growth using the combination ratio described by Dings et al. [38]. Fractional tumor volume (FTV) was first determined as follows:

FTV = (TV_f, treated_)/(TV_f, control_),

where TV_f_ is the average tumor volume at the end of the study. Then, the combination effect was evaluated by calculating the combination ratio as follows:

Combination ratio = (expected FTV)/(observed FTV),

where expected FTV = (FTV_AspA_) × (FTV_Irinotecan_), and observed FTV = (TV_f, combination_) / (TV_f, control_). A ratio of >1 indicates synergism, whereas a ratio of <1 indicates a less than additive effect.

### 4.11. Immunohistochemistry

The xenograft tumor tissues were fixed in 4% paraformaldehyde (PFA) and embedded in paraffin. The paraffin-embedded sections were serially deparaffinized, rehydrated, and subjected to antigen retrieval. The sections were incubated with Ki-67 antibody (Dako, Glostrup, Denmark), detected using the LSAB^TM^ + System-HRP kit (Dako) and counterstained with hematoxylin. The stained sections were observed and photographed with a microscope.

### 4.12. Statistical Analysis

Data from the in vivo study are presented as the means ± SD. The statistical significance (*P* < 0.05) was assessed using Student’s *t*-test. All statistical tests were two-sided.

## 5. Patents

Shin, J.; Oh, K.-B.; Oh, D.-C.; Lee, S.K.; Liao, L.; You, M.J. Novel peptide compound, production method therefor, and use thereof. KR 20160047976 A 20160503, 2016; PCT WO 2016064245 AI.

## Figures and Tables

**Table 1:** Inhibition of cancer cell proliferation by asperphenins and synthetic derivatives.

Compounds	IC_50_ (µM), 72 h			
	RKO	SNU638	SK-HEP-1	MDA-MB-231
Asperphenin A (**1**)	0.84 ± 0.26	4.31 ± 0.83	2.89 ± 0.03	6.48 ± 0.74
Asperphenin B (**2**)	1.26 ± 0.43	7.59 ± 1.77	3.08 ± 0.18	9.43 ± 0.39
Cycloasperphenin A (**3**)	>50	>50	>50	>50
Cycloasperphenin B (**4**)	>50	>50	>50	>50
7-Hydroxyasperphenin A (**5**)	24.23 ± 1.05	42.55 ± 4.59	37.58 ± 0.96	>50
7-*epi*-Hydroxyasperphenin A (**6**)	28.12 ± 4.36	49.26 ± 0.93	40.93 ± 0.66	>50
7-Hydroxyasperphenin B (**7**)	36.32 ± 1.45	42.40 ± 1.27	48.09 ± 0.01	>50
7-*epi*-Hydroxyasperphenin B (**8**)	27.75 ± 2.98	>50	>50	>50
7,15(*S*)-Dihydroxyasperphenin A (**9**)	>50	>50	>50	>50
7,15(*S*)-Dihydroxyasperphenin B (**10**)	>50	>50	>50	>50
7,15(*R*)-Dihydroxyasperphenin B (**11**)	>50	>50	>50	>50
Etoposide ^1^	3.82 ± 0.74	0.30 ± 0.05	0.49 ± 0.12	10.72 ± 0.88

^1^ Etoposide was used as a positive control.

**Table 2:** Combination index values and relevant descriptions on the co-treatment of AspA and irinotecan.

Irinotecan (μM)	CI Value	Description
1.25	0.811 ± 0.204	Moderate synergism
2.5	0.756 ± 0.100	Moderate synergism
5	0.694 ± 0.096	Synergism
10	0.652 ± 0.087	Synergism

**Table 3:** Combination index values and relevant descriptions on the co-treatment of AspA and paclitaxel (TXL).

TXL (nM)	CI Value	Description
0.16	1.673 ± 0.106	Antagonism
0.8	1.925 ± 0.305	Antagonism
4	0.742 ± 0.086	Moderate synergism
20	1.185 ± 0.102	Slight antagonism

**Table 4:** Inhibition of RKO tumor xenograft growth in nude mice.

Group	AspA (mg/kg)		4 mg/kg of Irinotecan	Combination
	4	8		
Inhibition rate (%)	38.9 ± 24.5	68.7 ± 17.1	70.0 ± 11.3	86.9 ± 11.9

**Table 5:** Synergistic effect of combined treatment with AspA and irinotecan in RKO cell-implanted xenografts.

FTV ^1^		Expected FTV	Observed FTV	Combination Ratio
4 mg/kg of AspA	4 mg/kg of Irinotecan			
0.61	0.30	0.18	0.13	1.39

^1^ FTV: Fractional tumor volume.

## Supplementary Materials

The following are available online at https://www.mdpi.com/1660-3397/18/2/110/s1, Figure S1: Effect of asperphenins and synthetic derivatives on the growth of human cancer cell lines.
