# Supplementary material for: Antitumor Activity of Asperphenin A, a Lipopeptidyl Benzophenone from Marine-Derived Aspergillus sp. Fungus, by Inhibiting Tubulin Polymerization in Colon Cancer Cells
Source: Mar Drugs. 2020 Feb 13;18(2):110. doi: 10.3390/md18020110 (PMC7073961; doi:10.3390/md18020110)
Supplement: Supplementary file 1 [file marinedrugs-18-00110-s001.pdf]

Anti-tumor activity of Asperphenin A, a lipopeptidyl benzophenone from marine-derived *Aspergillus* sp. fungus, by inhibiting tubulin polymerization in colon cancer cells

Song Yi Bae [1], Lijuan Liao [1], So Hyun Park [1], Won Kyung Kim [1], Jongheon Shin [1,*] and Sang Kook Lee [1,*]

[1] College of Pharmacy, Natural Products Research Institute, Seoul National University, Seoul 08826, Republic of Korea; baesy722@gmail.com (S.Y.B.); liao_0909@126.com (L.L.); hanirela@snu.ac.kr (S.H.P.); dnjsrud6764@naver.com (W.K.K.)

* Correspondence: shinj@snu.ac.kr (J.S.); sklee61@snu.ac.kr (S.K.L.); Tel.:+82-2-880-2484(J.S.); +82-2-880-2475(S.K.L.)

Supplementary Materials

Figure S1: Effect of asperphenins and synthetic derivatives on the growth of human cancer cell lines.

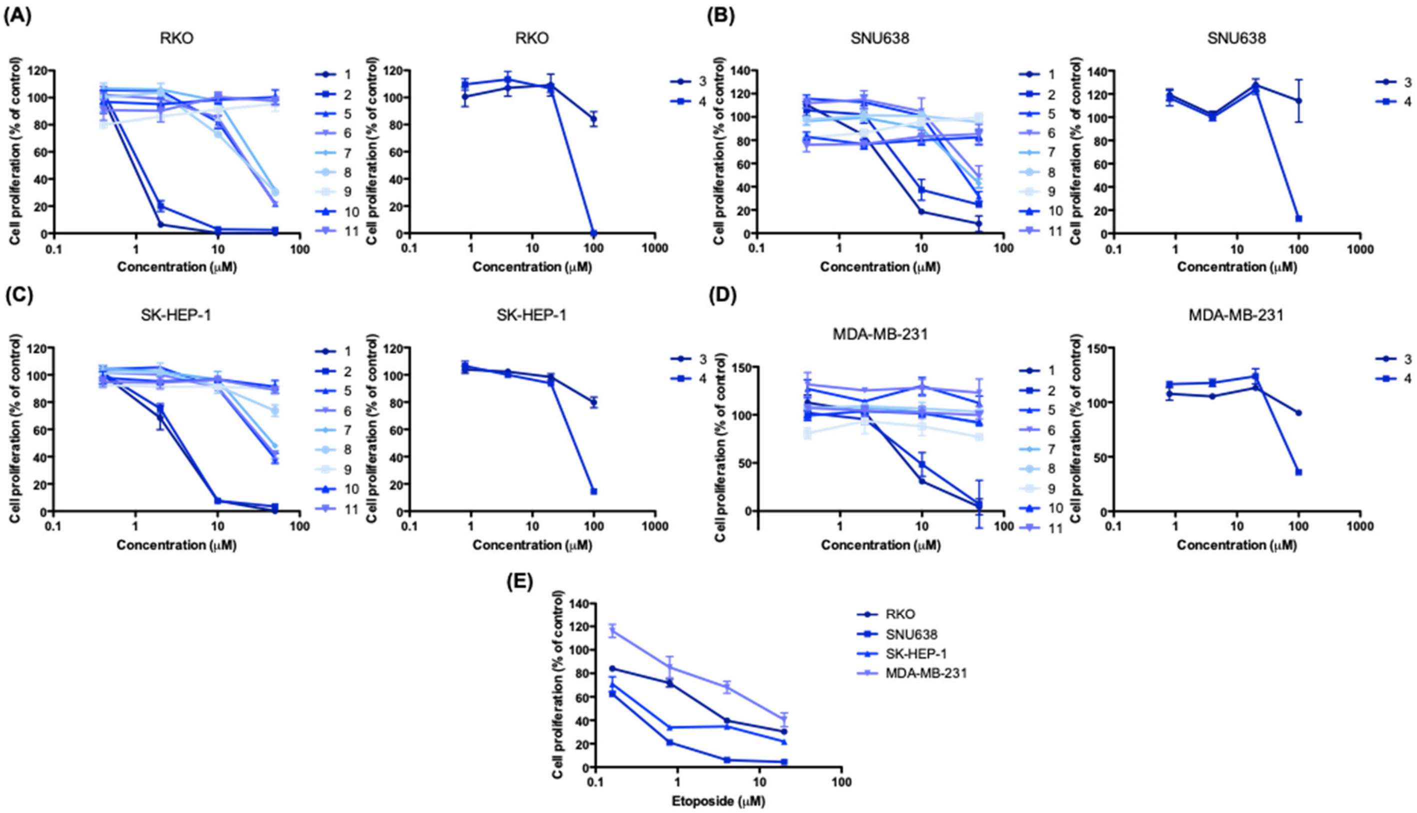

Figure S1. Effect of asperphenins and synthetic derivatives on the growth of human cancer cell lines. (**A-E**) RKO (A), SNU638 (B), SK-HEP-1 (C) and MDA-MB-231 (D) cells were treated with the indicated compounds or etoposide (E) for 72 h, and then the cell proliferation was measured by SRB assay. Etoposide was used as a positive control. The data are presented as the means ± SD. Asperphenin A (1); asperphenin B (2); cycloasperphenin A (3); cycloasperphenin B (4); 7-hydroxyasperphenin A (5); 7-*epi*-hydroxyasperphenin A (6); 7-hydroxyasperphenin B (7); 7-*epi*-hydroxyasperphenin B (8); 7, 15(*S*)-dihydroxyasperphenin A (9); 7,15(*S*)-dihydroxyasperphenin B (10); 7,15(*R*)- dihydroxyasperphenin B (11).
